# *Rehmannia glutinosa* Polysaccharides: Optimization of the Decolorization Process and Antioxidant and Anti-Inflammatory Effects in LPS-Stimulated Porcine Intestinal Epithelial Cells

**DOI:** 10.3390/antiox12040914

**Published:** 2023-04-12

**Authors:** Heng Ren, Kejie Li, Yan Min, Binhang Qiu, Xiaolu Huang, Jingxin Luo, Liwen Qi, Maoli Kang, Peng Xia, Hanzhen Qiao, Jun Chen, Yaoming Cui, Liping Gan, Peng Wang, Jinrong Wang

**Affiliations:** 1 School of Bioengineering, Henan University of Technology, Lianhua Street, Hi-Tech Zone, Zhengzhou 450000, China; 2 Jiangxi Province Key Laboratory of Animal Nutrition, Jiangxi Province Key Innovation Center of Integration in Production and Education for High-Quality and Safe Livestock and Poultry, Jiangxi Agricultural University, Nanchang 330045, China

**Keywords:** *Rehmannia glutinosa* polysaccharides, decolorization, antioxidant, anti-inflammatory, pathways

## Abstract

Polysaccharide decolorization has a major effect on polysaccharide function. In the present study, the decolorization of *Rehmannia glutinosa* polysaccharides (RGP) is optimized using two methods—the AB-8 macroporous resin (RGP-1) method and the H_2_O_2_ (RGP-2) method. The optimal decolorization parameters for the AB-8 macroporous resin method were as follows: temperature, 50 °C; macroporous resin addition, 8.4%; decolorization duration, 64 min; and pH, 5. Under these conditions, the overall score was 65.29 ± 3.4%. The optimal decolorization conditions for the H_2_O_2_ method were as follows: temperature, 51 °C; H_2_O_2_ addition, 9.5%; decolorization duration, 2 h; and pH, 8.6. Under these conditions, the overall score was 79.29 ± 4.8%. Two pure polysaccharides (RGP-1-A and RGP-2-A) were isolated from RGP-1 and RGP-2. Subsequently, their antioxidant and anti-inflammatory effects and mechanisms were evaluated. RGP treatment activated the Nrf2/Keap1 pathway and significantly increased the activity of antioxidant enzymes (*p* < 0.05). It also inhibited the expression of pro-inflammatory factors and suppressed the TLR4/NF-κB pathway (*p* < 0.05). RGP-1-A had a significantly better protective effect than RGP-2-A, likely owing to the sulfate and uronic groups it contains. Together, the findings indicate that RGP can act as a natural agent for the prevention of oxidation and inflammation-related diseases.

## 1. Introduction

Farmed pigs constantly face various health challenges, such as weaning stress [1], fungal poisoning [2], and endotoxin stress [3]. Several of these conditions exert intestinal effects, leading to the excessive production of pro-inflammatory cytokines and reactive oxygen species (ROS). This results in further intestinal inflammation and oxidative stress (OS) [4,5,6], which cause epithelial damage in intestinal tissues, promoting pathogen entry and transmission [7]. A well-functioning gut is critical for the prevention of gastrointestinal diseases. Thus, feed supplemented with appropriate antioxidants can improve and maintain intestinal health. In turn, better intestinal barrier function can help in preventing pathogen invasion and maintaining intestinal homeostasis [8,9].

*Rehmannia glutinosa* (RG) is a rhizome plant from Radix Rehmanniae, and it was first described in Sheng Nong’s herbal classic. RG, both fresh and dried, is edible and has been used in traditional Chinese medicine for over 2000 years. It is widely cultivated in the provinces of Henan, Shandong, and Shanxi [10,11]. So far, polysaccharides, alkaloids, terpenes, flavonoids, and other active compounds have been isolated from RG. These compounds have shown antioxidant [12], anti-inflammatory [13], antitumor [14,15], antiviral [16], immune-enhancing [10,17], and antiglycemic effects [18], among other benefits. RG and its extracts have been widely used as nutritional and health supplements as well as pharmaceutical products for both humans and animals [13].

Polysaccharides—biopolymers composed of several monosaccharides connected by glycosidic bonds—are the main active functional components of RG. Polysaccharides are typically extracted and purified from the roots and stems of RG and have a relative molecular weight of 4 × 10^3^ to 5 × 10^4^ Da [19,20]. Owing to their excellent antioxidant [20], anti-inflammatory [15], and immune-enhancing [21] properties, polysaccharides from RG (RGP) have attracted immense attention. At present, several commercially available human and animal health care products, functional foods, cosmetics, and drugs contain polysaccharides as active ingredients. However, plant tissues are often naturally rich in pigments, which interfere with the extraction and purification of polysaccharides. If pigments are not removed properly, the subsequent analysis of polysaccharide structure and function is seriously affected. In addition, during the process of polysaccharide separation and purification, column fillers often get contaminated, significantly increasing production costs [22]. Thus, decolorization methods are applied for polysaccharide purification. 

At present, polysaccharide decolorization is mainly conducted using physical and chemical methods. Physical decolorization processes typically use macroporous resin [23] and activated carbon [24], whereas H_2_O_2_ is the main agent for chemical decolorization [22]. Previously [20], we obtained two types of decolorized RGP using AB-8 macroporous resin and H_2_O_2_ (RGP-1-A and RGP-2-A, respectively) and analyzed their physicochemical and structural properties. Our study showed that the uronic acid contents of RGP-1-A and RGP-2-A were 19.02 ± 0.42% and 1.1 ± 0.27%, and their sulfuric acid group contents were 11.83 ± 0.80% and 8.56 ± 1.20%, respectively. The molar ratio of glucose:galactose:arabinose:galacturonic acid:rhamnose was 15.6:8.9:3.6:4.7:1 in RGP-1-A and 31.8:47.2:2.0:1 in RGP-2-A. The molecular weights of RGP-1-A and RGP-2-A were determined using gel permeation chromatography and were found to be 18,964 and 3305 Da, respectively. X-ray diffraction showed that both RGP-1-A and RGP-2-A had amorphous structures, while thermogravimetric analysis demonstrated that RGP-2-A had better thermal stability than RGP-1-A. Scanning electron microscopy revealed that RGP-1-A was mainly shaped like irregular and large curly flakes, while RGP-2-A formed small flakes. 

Another previous study investigated the physicochemical and structural properties of RGP decolorized using two different methods [20]. The structure–activity relationship of these RGP was analyzed, and their scavenging abilities against DPPH, ABTS, and hydroxyl radicals were determined in vitro. The results indicated that pigment removal is essential for the structural analysis and functional evaluation of RGP. Although some reports have demonstrated that RGP can alleviate oxidative damage in murine myocardial cells [25,26,27], the mechanism via which RGP alleviates injury in piglet intestinal epithelial cells remains unclear.

Therefore, in this study, we have used a single-factor and response surface method (RSM) design to optimize the decolorization process of RGP. In addition, we used an LPS-induced IPEC-1 cell injury model to investigate the regulatory effects of RGP on the Keap1/Nrf2 and NF-κB/TLR4 signaling pathways and to elucidate the molecular mechanism through which RGP alleviates IPEC-1 cell injury. This study provides data-based evidence and a theoretical basis for the development and application of RGP in animal health products.

## 2. Materials and Methods

Raw RG materials were bought from the Chinese Wen County Agricultural Products Market. IPEC-J2 cells were purchased from BLUEFBIO Bio-Tech Co. (Shanghai, China). AB-8 macroporous resin, H_2_O_2_, and sulfuric acid were purchased from Kemiou Chemical Reagent Co., Ltd. (Tianjin, China). Dulbecco’s modified Eagle medium:nutrient mixture F-12 (DMEM/F12), phosphate-buffered saline (PBS), penicillin–streptomycin, LPS, dimethyl sulfoxide (DMSO), and thiazolyl blue tetrazolium bromide (MTT) were procured from Solarbio Tech Co. (Beijing, China). All chemical reagents used in this study were of analytical grade.

### 2.1. Preparation of RGP

A previously described experimental technique was used for RGP extraction [20]. RG rhizomes were selected, cleaned, and cut thinly. The slices were air-dried at 60 °C. To generate a rough RG powder, the slices were crushed and passed through a 40-mesh filter. Then, 40 g of the powdered sample was combined with 500 mL of ultrapure water and subjected to 200 W ultrasonic treatment at 65 °C for 1 h. The solution obtained was filtered after centrifugation for 10 min (5000× *g*). After being concentrated, the supernatant was precipitated with anhydrous ethanol and kept at 4 °C for 12 h. Crude RGP was obtained by re-dissolving the precipitate in ultrapure water and freeze-drying. For decolorization studies, crude RGP was dissolved in deionized water to create an 8 mg/mL polysaccharide solution.

### 2.2. Calculation of Decolorization Index

#### 2.2.1. Decolorization Rate (DR)

A previously described approach was used to calculate the DR of RGP, with slight modifications [23,28]. An un-decolorized polysaccharide solution was scanned across the full UV spectrum, and the maximum absorption peak was found at 404 nm. In order to determine the DR (1), the absorbance of the polysaccharide solution at 404 nm was evaluated both before and after decolorization.

(1) $$\mathrm{DR}\;\left(\%\right)=\frac{\left(A_{1}-A_{2}\right)}{A_{1}}\times 100\%$$

where A_1_ is the absorbance of the RGP solution before decolorization, and A_2_ is the absorbance of the RGP solution after decolorization.

#### 2.2.2. Retention Rate (RR)

The total sugar content was used to compute the RR of polysaccharides after decolorization. The total sugar content in the RGP solution before and after decolorization was measured using the phenol–sulfuric acid method [29]. An equation was then used to calculate the RR (2).

(2) $$\mathrm{RR}\;\left(\%\right)=\frac{A_{2}}{A_{1}}\times 100\%$$

where A_1_ and A_2_ are the absorbances of the RGP solution at 490 nm before and after decolorization, respectively.

#### 2.2.3. Composite Score (CS)

The overall CS was calculated using a weighted method, with a weight of 0.5 for both polysaccharide decolorization and polysaccharide retention. The CS was calculated using Equation (3).

(3) CS (%)=0.5×DR+0.5×RR

### 2.3. Optimization of RGP Decolorization Using AB-8 Macroporous Resin

#### 2.3.1. Single-Factor Experiment (SFE) for RGP Decolorization Using AB-8 Macroporous Resin

SFEs were used to examine the decolorization of polysaccharides at different values of AB-8 macroporous resin addition (4%, 6%, 8%, 10%, and 12%), treatment durations (20, 40, 60, 80, and 100 min), temperatures (35, 40, 45, 50, 55, and 60 °C), and pH values (4, 5, 6, 7, 8, and 9). The impact of various conditions was determined based on the DR and RR.

#### 2.3.2. RSM for RGP Decolorization Using AB-8 Macroporous Resins

Using the Box-Behnken design (BBD) of the RSM for process optimization—and AB-8 macroporous resin addition (A, %), temperature (B, °C), duration (C, min), and pH (D) as independent variables—the resultant composite score (CS) was calculated. The levels and codes of the AB-8 macroporous resin decolorization variables used in the BBD are shown in Table 1. The entire experiment, consisting of 27 runs, was performed in the specific order described in Table S1.

### 2.4. Optimization of RGP Decolorization Using H_2_O_2_

#### 2.4.1. SFE for RGP Decolorization Using H_2_O_2_

SFEs were conducted to determine the best temperature (35, 40, 45, 50, 55, and 60 °C), duration (20, 40, 60, 80, and 100 min), pH (4, 5, 6, 7, 8, and 9), and H_2_O_2_ addition (1.0%, 1.5%, 2.0%, 2.5%, and 3.0%) for RGP decolorization. The impact of various conditions was determined based on DR and RR.

#### 2.4.2. RSM for RGP Decolorization Using H_2_O_2_

Using the BBD of the RSM for process optimization—and temperature (A, °C), duration (B, h), pH (C), and H_2_O_2_ addition (D, %) as independent variables—the resultant CS was calculated. The levels and codes of the H_2_O_2_ decolorization variables used in the BBD are shown in Table 2. The entire experiment consisting of 27 runs was performed in the specific order illustrated in Table S2.

### 2.5. Purification of RGP

We purified the two RGP based on our previous study [20]. The specific process was as follows. RGP decolorized with AB-8 macroporous resin (RGP-1) and H_2_O_2_ (RGP-2) were deproteinized using the Sevag method. The solution was dialyzed in ultrapure water using a dialysis bag with a Mw cut-off of 3500 Da. The sample solution was then freeze-dried to obtain the two RGP samples (RGP-1/RGP-2). Then, RGP-1/RGP-2 (0.1 g) was dissolved in 10 mL of ultrapure water and filtered through a 0.45-μm microporous membrane. The RGP samples were loaded onto a 2.6 × 40 cm DEAE-52 cellulose column. Different fractions were eluted sequentially using different concentrations of NaCl dissolved in ultrapure water (0.1, 0.3, 0.5, 1 mol/L). The maximum elution fraction of RGP-1/RGP-2 was dialyzed in ultrapure water using a dialysis bag with a Mw cut-off value of 3500 Da to remove small molecular impurities. It was concentrated at 65 °C and freeze-dried to obtain the final polysaccharides (RGP-1-A/RGP-2-A). RGP-1-A and RGP-2-A were further purified using Sephadex G-100 columns (2.6 × 40 cm, Yuanye Bio-Technology, Shanghai, China). Polysaccharides were eluted with 0.5 mL/min ultrapure water and collected in separate tubes. Then, the polysaccharide content in each tube was analyzed using the phenol–sulfuric acid method, and the concentration curve was drawn. The tube corresponding to the elution peak was selected. Subsequently, the solution was concentrated under low pressure and freeze-dried to obtain the pure polysaccharides RGP-1-A and RGP-2-A.

### 2.6. Protective Effect of RGP on IPEC-J2 Cells

#### 2.6.1. Cell Culture

IPEC-J2 cells were cultured in DMEM containing 10% FBS and 1% penicillin–streptomycin at 37 °C under 5% CO_2_.

#### 2.6.2. Viability Assay

MTT assays were used to assess cell viability after RGP treatment [30]. In 96-well plates, IPEC-J2 cells were cultured for 12 h at a density of 5 × 10^3^ cells/well. DMEM containing different concentrations of RGP-1-A or RGP-2-A (ranging from 0 to 800 μg/mL) was used to pretreat cells for 24 h, and cells were then treated with DMEM containing LPS (0, 0.1, 1, 10, and 100 μg/mL) for 24 h. We added 20 μL of MTT to each well before incubating the cells at 37 °C for 3 h. Then, the medium was discarded, and 150 μL of DMSO was added to each well. Absorbance was measured at 490 nm after 10 min following complete dissolution. Cells cultured without RGP and LPS treatment were considered controls. Cell viability was calculated using the following equation.

$$\mathrm{Cell}\;\mathrm{viability}\left(\%\right)=\frac{A_{1}-A_{0}}{A_{2}-A_{0}}\times 100\%$$

where A_0_ is the absorbance of the blank wells, A_1_ is the absorbance of the treatment group, and A_2_ is the absorbance of the control group.

#### 2.6.3. Determination of ROS Levels

Flow cytometry was utilized to measure intracellular ROS levels per the instructions of a commercial kit (Beyotime Biotech, Shanghai, China). IPEC-J2 cells were cultured in 6-well plates at a density of 1 × 10^5^/well for 12 h. RGP (100, 200, and 400 μg/mL) was added at different concentrations for 24 h, except in the control and model groups. This was followed by LPS treatment (10 μg/mL) for 24 h (all wells except control). The culture medium was discarded after treatment, and the cells were washed in PBS. The production of ROS in IPEC-J2 cells was estimated using a ROS measuring kit (Beyotime Biotech, Shanghai, China). DCFH-DA was diluted to 1:1000 (*v*/*v*, mL/mL) in serum-free medium. Cells were incubated with 1 mL of DCFH-DA in a cell incubator at 37 °C for 20 min. ROS levels were analyzed using a BD LSRFortessa flow cytometer (BD Company, Franklin Lake, NJ, USA).

#### 2.6.4. Determination of Mitochondrial Membrane Potential (MMP) 

MMP was determined using the JC-1 test kit (Beyotime Biotech, Shanghai, China) according to the manufacturer’s instructions. Briefly, IPEC-J2 cells were treated using the experimental design described previously. The cells were then incubated with 1 mL of the JC-1 working fluid for 30 min at 37 °C in a cell incubator. The supernatant was removed after incubation. The cells were washed twice with JC-1 buffer and then analyzed using a BD LSRFortessa flow cytometer (BD Company, Franklin Lake, NJ, USA).

#### 2.6.5. Apoptosis Assay

Annexin V-FITC/PI (Beyotime Biotechnology, Shanghai, China) was used to detect apoptosis activity using flow cytometry. Cells were treated according to the experimental design described previously. This was followed by two washes in PBS, digestion with trypsin, and resuspension in PBS. Then, 100 μL of the cell suspension was transferred to a 2-mL EP tube, and the cell density was increased to 1 × 10^6^ cells/mL. The supernatant was discarded after 5 min of centrifugation at 1000× *g*. The cells were gently resuspended in a mixture of 195 μL Annexin V-FITC conjugate, 5 μL Annexin V-FITC, 10 μL PI solution, and 400 μL PBS. After 20 min of incubation at 37 °C away from light, apoptosis was analyzed using the BD LSRFortessa flow cytometer (BD Company, Franklin Lake, NJ, USA).

#### 2.6.6. Evaluation of Oxidation-Related Indexes 

Cells were treated according to the experimental design described above. RIPA lysis solution was used to collect the protein supernatant from cells. Malondialdehyde (MDA, Beyotime Biotech, China, Shanghai) and superoxide dismutase (SOD, Beyotime Biotech, Shanghai, China) levels in the cells were measured using commercial kits.

#### 2.6.7. RT-PCR Analysis

IPEC-J2 cells were treated as described above. The RNAiso plus reagent was used to extract the total RNA after treatment (Takara bio, Beijing, China). A NanoDrop 2000 instrument (Thermo Scientific, Waltham, MA, USA) was used to calculate the quantity and quality of RNA. A reverse transcription kit (Takara bio, Japan) was used to synthesize cDNA. For RT-PCR analysis, SYBR qPCR Master Mix (Vazyme, Nanjing, China) and the qTOWER 3G RT-PCR System (Analytik Jena, Thuringia, Germany) were used. Standard curves for each primer were obtained using 10-fold serial dilutions of the reference cDNA. Real-time PCR efficiencies (E) were calculated according to the following equation: E = 10^[−1/slope]^. Data were normalized according to GAPDH expression. Tables S3 and S4 list the primer sequences and PCR parameters, respectively. The relative levels of gene expression were calculated using the 2^−ΔΔCt^ technique.

### 2.7. Statistical Analysis

All values are expressed as the mean ± standard deviation (SD). Statistical analyses were performed using one-way ANOVA with SPSS Statistics 20.0 software (IBM, Armonk, NY, USA). *p* < 0.05 was considered statistically significant.

## 3. Results

### 3.1. Optimization of RGP Decolorization Using AB-8 Macroporous Resin

#### 3.1.1. Model Fitting

**Figure 1:**
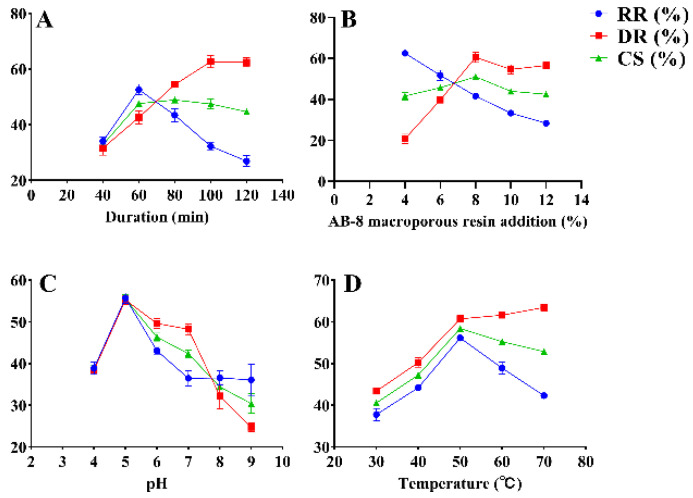
SFEs for the decolorization of RGP using AB-8 macroporous resin. DR: decolorization rate (%); RR: retention rate (%); CS: composite score (%). (**A**–**D**): duration, AB-8 macroporous resin addition, pH, temperature.

The effects of decolorization temperature, AB-8 macroporous resin addition, decolorization duration (duration), and pH on RGP decolorization were investigated using SFEs. The results are shown in Figure 1. The initial decolorization duration was set to 40, 60, 80, 100, and 120 min, and other conditions were fixed at a resin addition of 20%, pH of 7, and decolorization temperature of 60 °C. The RR and CS significantly increased as time increased and peaked (52.61% ± 1.57% and 47.62% ± 0.37%, respectively) at 60 min (Figure 1A). Nevertheless, the RR and CS decreased when the decolorization duration became greater than 60 min. However, the DR increased as time increased and reached 42.64% ± 1.94% when the decolorization duration was 60 min. Thus, 60 min was selected as the optimal decolorization duration. The DR and CS increased from 20.73 ± 1.85% and 41.62 ± 1.52% to 60.49% ± 2.05% and 51.05% ± 0.55%, respectively, as the addition of AB-8 macroporous resin increased from 4% to 8% (Figure 1B). The DR and CS peaked when the addition of resin was 8% and decreased thereafter. Meanwhile, the RR decreased with the addition of resin, reaching 41.62% ± 1.06% at 8% resin addition. Ultimately, 8% resin addition was selected to achieve a high efficiency of decolorization. The pH was set to 4, 5, 6, 7, 8, and 9 at a constant temperature of 60 °C (Figure 1C). The RR, DR, and CS peaked (55.73% ± 0.99%, 55.25% ± 0.91%, and 55.49% ± 0.89%, respectively) at a pH of 5. When the pH increased from 5 to 9, the RR, DR, and CS decreased significantly. The RR, DR, and CS increased with temperature and peaked at 50 °C (56.15% ± 0.12%, 60.71% ± 0.65%, and 58.43% ± 0.27%, respectively) (Figure 1D). Meanwhile, a temperature greater than 50 °C resulted in a decline in DR and CS. Therefore, the optimal temperature of 50 °C was selected for decolorization.

Then, the effects of the four parameters on the efficacy of decolorization were examined using the RSM and BBD. The experimental data were examined using multiple regression analysis, which yielded the equation below.

$$\begin{array}{cc} Y= & 70.74-0.5012A-0.2745B+0.257C+0.6061D+0.0355\mathrm{AB}-0.3201\mathrm{AC}\\ & -1.09\mathrm{AD}+0.6258\mathrm{BC}-0.1393\mathrm{BD}-1.52\mathrm{CD}-2.23A^{2}\\ & -1.77B^{2}-1.09C^{2}-2.19D^{2} \end{array}$$

As shown in Table 3, the F-value (53.27) and *p*-value (<0.0001) of the model indicated that the model was significant. Moreover, the adjusted *R*^2^ (0.9497) revealed that the model had a high level of significance. Only 2.32% of the total variation could not be explained by the model, according to the *R*^2^ value of 0.9768. Additionally, the quadratic terms (A^2^, B^2^, C^2^, and D^2^), some interaction terms (AD, BC, and CD), and linear coefficients (A, B, C, and D) were significant (*p* < 0.05), demonstrating that these factors could significantly affect RGP decolorization. The *p*-value (0.2995) and F-value (2.71) for the lack of fit were not significant, indicating that the model was credible. The coefficient of variation (CV), which signifies the similarity between the predicted and experimental values, was 0.5404. This demonstrated that the model was highly reliable.

#### 3.1.2. Optimization of Decolorization Conditions

The interactions between factors and responses can be displayed using two- and three-dimensional contour plots, respectively. The shape of the contour plot—circular or elliptical—indicates the significance of the reciprocal interactions between variables. An elliptical contour plot indicates a substantial interaction between the respective variables, while a circular plot reveals a minor interaction between the variables. In this study, the results of the combined RGP decolorization score—which was affected by decolorization temperature, AB-8 macroporous resin addition, decolorization duration, and pH—are presented in Figure S1. The contour plots seen in Figure S1A,B are circular, demonstrating that the interaction between the decolorization temperature and the addition of AB-8 macroporous resin was negligible. The contour plots shown in Figure S1C,D are also circular, demonstrating that the interaction between decolorization duration and the quantity of AB-8 macroporous resin addition was not significant. The contour plots shown in Figure S1E,F are oval, indicating the significance of the interaction between the decolorization temperature and the quantity of the AB-8 macroporous resin supplied. The contour plots shown in Figure S1G,H are elliptical, revealing the significance of the interplay between decolorization duration and temperature. The contour plots shown in Figure S1I,J are circular, demonstrating that the relationship between decolorization temperature and pH was not significant. The contour plots shown in Figure S1K,L are elliptical, demonstrating the importance of the interplay between decolorization duration and pH.

The optimal parameters obtained from the above experiments are as follows: decolorization temperature, 50.71 °C; AB-8 macroporous resin addition, 8.38%; decolorization duration, 63.83 min; and pH, 4.78. The maximum predicted CS of RGP under the optimized conditions was 69.15%. The actual conditions used for validating the results were as follows: decolorization temperature, 50 °C; AB-8 macroporous resin addition, 8.4%; decolorization duration, 64 min; and pH, 5.0. The actual CS was 65.29 ± 3.4%, consistent with the predicted value. 

### 3.2. Optimization of RGP Decolorization Using H_2_O_2_

#### 3.2.1. Model Fitting

**Figure 2:**
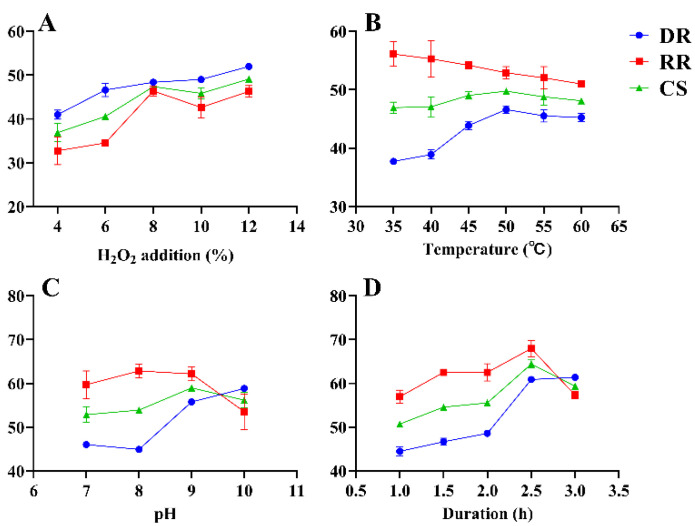
SFEs for the decolorization of RGP using H_2_O_2_. DR: decolorization rate (%); RR: retention rate (%); CS: composite score (%). (**A**–**D**): H_2_O_2_ addition, temperature, pH, duration.

The effects of decolorization temperature, decolorization duration, H_2_O_2_ addition, and pH on RGP decolorization were investigated using SFEs. The results are illustrated in Figure 2. The initial amount of H_2_O_2_ addition was set to 4%, 6%, 8%, 10%, and 12%. The other conditions were fixed at a temperature of 60 °C, pH of 9, and decolorization duration of 2 h. The RR, DR, and CS increased with an increase in H_2_O_2_ and peaked (46.37% ± 0.93%, 48.40% ± 0.37%, and 47.38% ± 0.65%, respectively) at 8% H_2_O_2_ addition (Figure 2A). With the increase in H_2_O_2_ addition beyond 8%, although the RR, DR, and CS increased slightly, the difference was not significant. Ultimately, 8% H_2_O_2_ was selected to achieve a high efficiency of decolorization. The effect of various decolorization temperatures (35, 40, 45, 50, 55, and 60 °C) on RR, DR, and CS is presented in Figure 2B. DR and CS peaked (46.62% ± 0.53%, 49.77% ± 0.33%, respectively) when the temperature was 50 °C and decreased at higher temperatures. RR tended to decrease as the temperature increased and reached 52.92% ± 0.87% when the temperature was 50 °C. Thus, 50 °C was selected as the optimal decolorization temperature. The pH values were set to 7, 8, 9, and 10 at a constant decolorization duration of 2 h (Figure 2C). When the pH increased from 7 to 9, RR and CS increased, and the values peaked at a pH of 9 (62.19% ± 1.30% and 58.99% ± 0.60%, respectively). However, pH values greater than 9 resulted in a reduced RR and CS. Therefore, the optimal pH of 9 was selected for decolorization. The effect of duration (1, 1.5, 2, 2.5, and 3 h) on decolorization is shown in Figure 2D. When the duration ranged from 1 to 2.5 h, the decolorization effect gradually increased with time. At a decolorization duration of 2.5 h, the RR was 67.94% ± 1.52%, DR was 60.88% ± 0.07%, and CS was 64.41% ± 0.76%. However, a decolorization duration greater than 2.5 h resulted in a decline in RR and CS. Therefore, the optimal duration of 2.5 h was selected for decolorization.

Then, the effects of the four parameters on the efficacy of decolorization were examined using the RSM and BBD. The experimental data were examined using multiple regression analysis, yielding the following equation.

$$\begin{array}{cc} Y= & 86.35+0.6895A+2.91B+0.5124C+1.19D+0.785\mathrm{AB}+1.29\mathrm{AC}\\ & +0.2575\mathrm{AD}+0.1575\mathrm{BC}-1.36\mathrm{BD}+1.77\mathrm{CD}-4.06A^{2}\\ & -3.41B^{2}-3.72C^{2}-2.69D^{2} \end{array}$$

As shown in Table 4, the F-value (10.03) and *p*-value (0.0001) of the model indicate that the model is significant. Moreover, the adjusted *R*^2^ (0.8294) shows that the model has a high level of significance. Only 8.87% of the total variation could not be explained by the model, as demonstrated by the *R*^2^ value of 0.9213. Additionally, the quadratic terms (A^2^, B^2^, C^2^, and D^2^) and some interaction terms (CD) and linear coefficients (B and D) are significant (*p* < 0.05), suggesting that these factors could significantly affect RGP decolorization. The *p*-value (0.5471) and F-value (0.7877) for the lack of fit are not significant, indicating that the model is credible. The CV is 1.77, demonstrating that the model is highly reliable.

#### 3.2.2. Optimization of Decolorization Conditions

Figure S2 shows the combined results of RGP decolorization CS values, as influenced by decolorization temperature, decolorization duration, H_2_O_2_ addition, and pH. The circular contour plots in Figure S2A,B and Figure S2C,D demonstrate that there was no significant interaction between decolorization temperature and decolorization duration or between decolorization temperature and pH, respectively. Similarly, the circular contour plots in Figure S2E,F and Figure S2G,F reveal that the interaction between the decolorization temperature and H_2_O_2_ addition and the interaction between decolorization duration and pH are both insignificant. The contour plots shown in Figure S2I,J are circular, demonstrating the lack of significance of the interaction between decolorization duration and H_2_O_2_ addition. Meanwhile, the elliptical contour plots in Figure S2K,L demonstrate the significance of the interplay between pH and H_2_O_2_ addition.

The optimal parameters obtained from the above experiments are: decolorization temperature of 51.06 °C, decolorization duration of 2.27 h, pH of 8.58, and H_2_O_2_ addition of 9.47%. Under the optimized conditions, the maximum predicted CS of RGP was 82.63%. However, the actual conditions for validating the results were a decolorization temperature of 51 °C, decolorization duration of 2 h, pH of 8.6, and H_2_O_2_ addition of 9.5%. The actual decolorization CS was 79.29 ± 4.8%, consistent with the predicted value.

### 3.3. Protection of IPEC-J2 Cells from LPS-Induced Inflammatory Damage

#### 3.3.1. Cytotoxicity Assay

**Figure 3:**
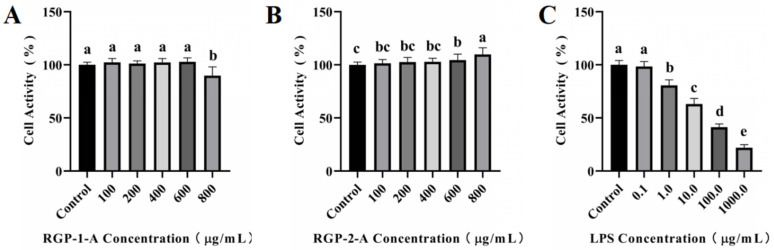
Cytotoxicity assay of RGP-1-A (**A**) and RGP-2-A (**B**) and LPS model establishment (**C**). RGP-1-A: decolorized pure polysaccharide of RGP obtained using AB-8 macroporous resin; RGP-2-A: decolorized pure polysaccharide of RGP obtained using H_2_O_2_. On the bar graphs, different lowercase letters signify a significant difference (*p* < 0.05).

As shown in Figure 3A,B, cytotoxicity analysis was performed using different concentrations of RGP-1-A and RGP-2-A (100–800 μg/mL). RGP-1-A was not cytotoxic at 100–600 μg/mL, but it exerted cytotoxic effects at 800 μg/mL, as evidenced by the inhibition of cell proliferation. Notably, no inhibitory effect was found in cytotoxicity assays for RGP-2-A.

As shown in Figure 3C, cell viability showed a significant decrease with an increase in LPS concentration. At 24 h, the cell viability decreased to 63.06% ± 5.31% at LPS concentrations of 10 μg/mL (versus untreated control cells). When the LPS concentration was increased to 100 μg/mL, the cell viability started to decrease to <50%. Hence, we chose an LPS concentration of 10 μg/mL for subsequent experiments.

#### 3.3.2. RGP Treatment Reduces the Levels of ROS in LPS-Induced IPEC-J2 Cells 

**Figure 4:**
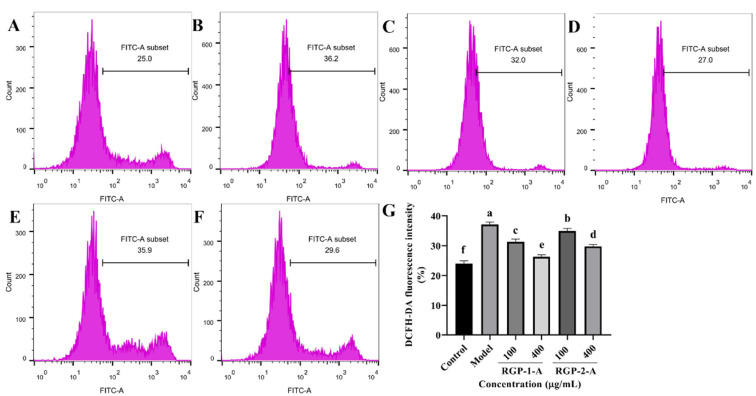
Effect of RGP pretreatment on ROS levels in LPS-induced IPEC-J2 cells. (**A**): Control group; (**B**): model group; (**C**): RGP-1-A low concentration; (**D**): RGP-1-A high concentration; (**E**): RGP-2-A low concentration; (**F**): RGP-2-A high concentration; (**G**):Quantitative analysis of ROS content in cells. RGP-1-A: decolorized pure polysaccharides of RGP obtained using AB-8 macroporous resin; RGP-2-A: decolorized pure polysaccharides of RGP obtained using H_2_O_2_. On the bar graphs, different lowercase letters signify a significant difference (*p* < 0.05).

ROS plays a key role in inflammation-induced diseases. Therefore, the current study has examined whether the ability of RGP to prevent ROS accumulation contributes to their protective effects. In comparison to the control treatment (Figure 4A), LPS treatment (Figure 4B) significantly increased ROS generation (*p* < 0.05). In comparison with the model group, the RGP–1–A (Figure 4C,D) and RGP–2–A (Figure 4E,F) treatment groups showed significantly lower intracellular ROS levels. Further, both effects were dose-dependent (*p* < 0.05). Notably, at the maximum concentration (400 μg/mL), intracellular ROS levels were significantly lower after RGP-1-A (Figure 4D) treatment than after RGP-2-A (Figure 4F) treatment (*p* < 0.05).

#### 3.3.3. RGP Treatment Attenuates the LPS-Induced Reduction in MMP

**Figure 5:**
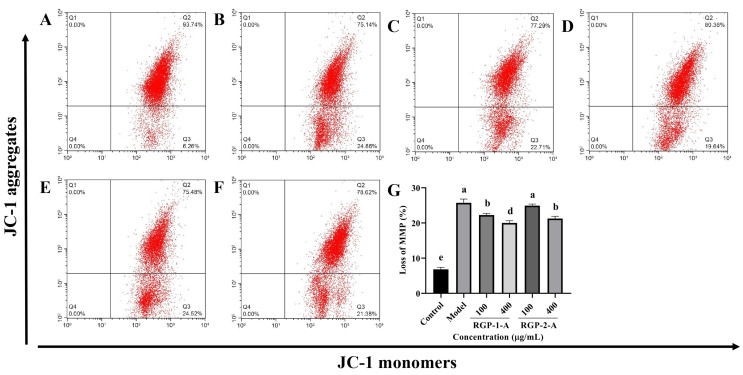
Effect of RGP pretreatment on MMP in LPS-induced IPEC-J2 cells. (**A**): control group; (**B**): model group; (**C**): RGP-1-A low concentration; (**D**): RGP-1-A high concentration; (**E**): RGP-2-A low concentration; (**F**): RGP-2-A high concentration; (**G**):Quantitative analysis of mitochondrial membrane potential changes. Q2 indicates the percentage of IPEC-J2 cells with high MMP, and Q3 indicates the percentage of IPEC-J2 cells with low MMP. RGP-1-A: decolorized pure polysaccharide of RGP obtained using AB-8 macroporous resin; RGP-2-A: decolorized pure polysaccharide of RGP obtained using H_2_O_2_. On the bar graphs, different lowercase letters signify a significant difference (*p* < 0.05).

The intrinsic apoptotic pathway is assumed to be largely dependent on the decrease in MMP. Figure 5A,B show that following LPS treatment, MMP decreased considerably in IPEC-J2 cells (*p* < 0.05). Both RGP-1-A and RGP-2-A could inhibit this LPS-induced MMP reduction in a dose-dependent manner (*p* < 0.05) (Figure 5B–F). However, at the highest dose (400 μg/mL), RGP-1-A had a substantially stronger inhibitory effect against the decrease in MMP than did RGP-2-A (*p* < 0.05).

#### 3.3.4. RGP Treatment Ameliorates LPS-Induced Apoptosis

**Figure 6:**
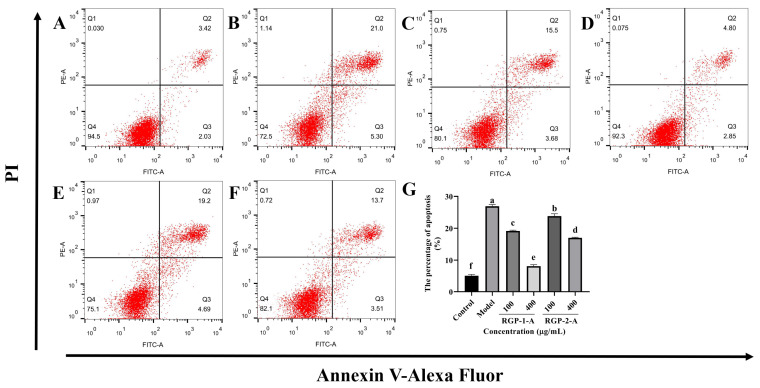
Effect of RGP pretreatment on apoptosis in LPS-induced IPEC-J2 cells. In (**A**–**F**), (**A**): control group; (**B**): model group; (**C**): RGP-1-A low concentration; (**D**): RGP-1-A high concentration; (**E**): RGP-2-A low concentration; (**F**): RGP-2-A high concentration; (**G**): Quantitative analysis of the number of apoptotic cells. Q1 represents the proportion of dead cells (necrosis), Q2 represents the proportion of late-apoptotic IPEC–J2 cells, Q3 represents the proportion of early-apoptotic IPEC–J2 cells, and Q4 represents the proportion of surviving IPEC–J2 cells. RGP-1-A: decolorized pure polysaccharides of RGP obtained using AB-8 macroporous resin; RGP-2-A: decolorized pure polysaccharides of RGP obtained using H_2_O_2_. On the bar graphs, different lowercase letters signify a significant difference (*p* < 0.05).

As shown in Figure 6, the fraction of apoptosis was significantly greater in the LPS-treated group (Figure 6B, 26.91% ± 0.53%) than in the control group (Figure 6A, 5.10% ± 0.43%) (*p* < 0.05). RGP-1-A (Figure 6C,D) and RGP-2-A (Figure 6E,F) had a significant inhibitory impact on LPS-induced apoptosis in IPEC-J2 cells (*p* < 0.05). The proportion of apoptosis was significantly lower in cells treated with a high dose (400 μg/mL) of RGP-1-A (Figure 6D, 8.12% ± 0.47%) than in cells treated with a high dose of RGP-2-A (Figure 6F, 17.01% ± 0.20%) (*p* < 0.05).

#### 3.3.5. RGP Treatment Ameliorates the Expression of Apoptosis-Related Genes Induced by LPS

**Figure 7:**
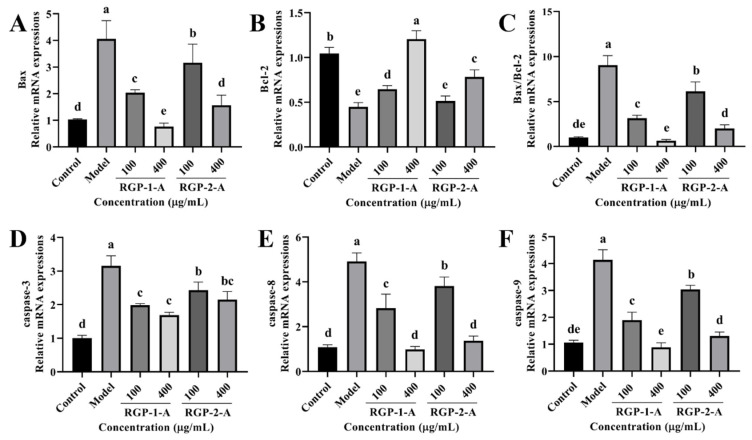
Effect of RGP on the relative expression of apoptosis-related genes in IPEC-J2 cells induced by LPS. (**A**), Bax; (**B**), Bcl-2; (**C**), Bax/Bcl-2; (**D**), caspase-3; (**E**), caspase-8; (**F**), caspase-9. RGP-1-A: decolorized pure polysaccharides of RGP obtained using AB-8 macroporous resin; RGP-2-A: decolorized pure polysaccharides of RGP obtained using H_2_O_2_. On the bar graphs, different lowercase letters signify a significant difference (*p* < 0.05).

Subsequently, we analyzed the activity of intracellular apoptotic pathways following RGP treatment based on mRNA levels. LPS significantly increased (*p* < 0.05) the relative mRNA expression of caspase-3, caspase-8, caspase-9, and Bax as well as the Bax/Bcl-2 ratio (Figure 7). However, RGP treatment reversed this effect. The relative levels of Bcl-2 mRNA were considerably lower in the LPS group than in the control group. However, these levels increased following RGP pretreatment. Overall, RGP-1-A was much more effective at inhibiting apoptosis than RGP-2-A.

#### 3.3.6. RGP Treatment Increases Intracellular Antioxidant Enzyme Activity 

**Figure 8:**
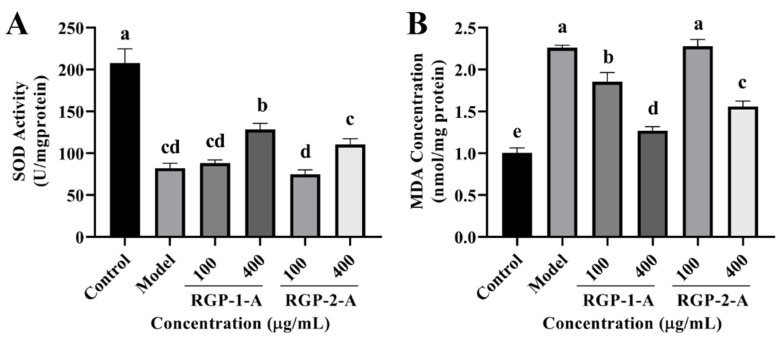
Effect of RGP on the antioxidant capacity of IPEC-J2 cells induced by LPS. (**A**), SOD; (**B**), MDA. RGP-1-A: decolorized pure polysaccharides of RGP obtained using AB-8 macroporous resin; RGP-2-A: decolorized pure polysaccharides of RGP obtained using H_2_O_2_. On the bar graphs, different lowercase letters signify a significant difference (*p* < 0.05).

To examine the antioxidant capacity of RGP, some oxidation-related indexes (SOD and MDA) were measured in IPEC-J2 cells. As shown in Figure 8A, RGP treatment considerably increased (*p* < 0.05) the quantity of SOD in cells, whereas LPS treatment markedly decreased (*p* < 0.05) these levels. Among the two RGPs, high-dose RGP-1-A had a stronger impact on SOD induction than high-dose RGP-2-A (*p* < 0.05). Following LPS treatment, cellular MDA levels were considerably higher than those in the control group (*p* < 0.05) (Figure 8B). However, MDA levels were considerably attenuated by RGP treatment (*p* < 0.05). Indeed, high-dose RGP-1-A had a stronger effect on MDA reduction than high-dose RGP-2-A (*p* < 0.05).

#### 3.3.7. RGP Treatment Promotes Antioxidant Signaling Pathways 

**Figure 9:**
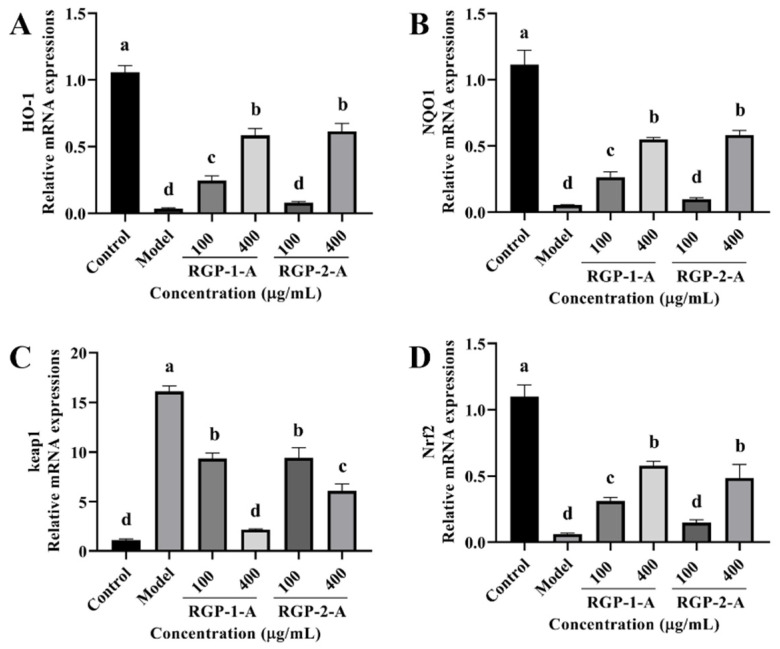
Effect of RGP pretreatment on the antioxidant enzyme content and relative mRNA expression of antioxidant pathway-related proteins in LPS-induced IPEC-J2 cells. (**A**), HO-1; (**B**), NQO1; (**C**), Keap1; (**D**), Nrf2 mRNA relative expression. RGP-1-A: decolorized pure polysaccharides of RGP obtained using AB-8 macroporous resin; RGP-2-A: decolorized pure polysaccharides of RGP obtained using H_2_O_2_. On the bar graphs, different lowercase letters signify a significant difference (*p* < 0.05).

Owing to these changes in antioxidant enzymes and lipid peroxidation levels, we examined the changes in intracellular antioxidant signaling pathways in our experimental groups. The relative expression of intracellular heme oxygenase-1 (HO-1), NQO1, and Nrf2 mRNAs was considerably reduced after LPS treatment when compared with the control (*p* < 0.05) (Figure 9A,B,D). However, this effect was reversed after RGP treatment (*p* < 0.05). As shown in Figure 9C, while Keap1 expression was dramatically increased following pretreatment with RGP, it was significantly lower in cells exposed to LPS (*p* < 0.05). Among the two polysaccharides, RGP-1-A was significantly better than RGP-2-A at upregulating intracellular antioxidant signaling pathways.

#### 3.3.8. RGP Treatment Inhibits Inflammatory Cytokines and Signaling Pathways

**Figure 10:**
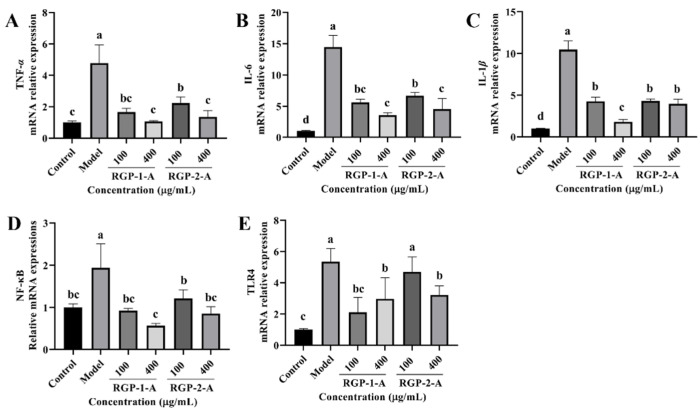
Effect of RGP pretreatment on the relative mRNA expression of inflammatory cytokines and signaling pathway proteins in LPS-induced IPEC-J2 cells. Figure (**A**−**E**) show the relative mRNA expression levels of TNF-α, IL-6, IL-1β, NF-κB, and TLR4, respectively. RGP-1-A: decolorized pure polysaccharides of RGP obtained using AB-8 macroporous resin; RGP-2-A: decolorized pure polysaccharides of RGP obtained using H_2_O_2_. On the bar graphs, different lowercase letters signify a significant difference (*p* < 0.05).

The levels of inflammatory cytokines (TNF-α, IL-6, and IL-1β) were significantly higher in the LPS group than in the control group (*p* < 0.05). Compared with the LPS group, the RGP groups showed significantly lower levels of inflammatory factors. Between the two polysaccharides, high-dose RGP-1-A had a significantly better effect on the reduction in intracellular inflammatory cytokines than high-dose RGP-2-A. Notably, the relative expression of TLR4 and NF-κB was significantly higher in the model group than in the control group, as demonstrated in Figure 10D,E (*p* < 0.05). In cells treated with RGP, the relative expression of TLR4 and NF-κB was markedly downregulated (*p* < 0.05). Cells treated with high doses of RGP-1-A showed a greater reduction in the relative expression of TLR4 and NF-κB than those treated with high doses of RGP-2-A (*p* < 0.05).

#### 3.3.9. RGP Treatment Attenuates LPS-Induced Barrier Dysfunction in IPEC-J2 Cells

**Figure 11:**
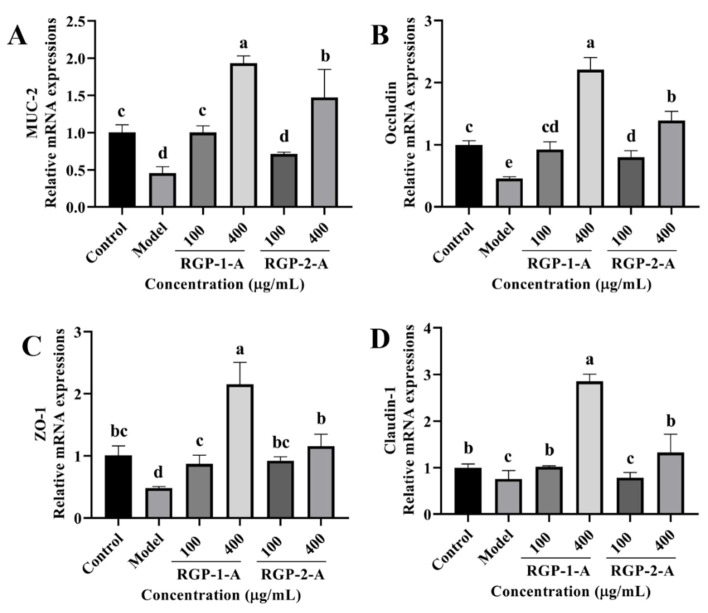
Effect of RGP on the relative mRNA expression of barrier-related proteins. (**A**): MUC-2, (**B**): occludin, (**C**): ZO-1, (**D**): claudin-1. RGP-1-A: decolorized pure polysaccharides of RGP obtained using AB-8 macroporous resin; RGP-2-A: decolorized pure polysaccharides of RGP obtained using H_2_O_2_. On the bar graphs, different lowercase letters signify a significant difference (*p* < 0.05).

We examined the mRNA levels of tight junction (TJ) proteins (occludin, claudin-1, and ZO-1) and MUC-2 to understand how RGP and LPS affect the intestinal barrier. As shown in Figure 11A–D, the model group had a lower expression of MUC-2, occludin, claudin-1, and ZO-1 than the control group (*p* < 0.05). However, RGP-treated cells showed a considerable increase in the relative expression of occludin, MUC-2, claudin-1, and ZO-1 (*p* < 0.05). Furthermore, high concentrations of RGP-1-A enabled better recovery of these mRNA levels than high concentrations of RGP-2-A (*p* < 0.05).

## 4. Discussion

Decolorization is a key step for the extraction and purification of plant polysaccharides. The yield and decolorization efficiency of polysaccharides can differ under different decolorization conditions. In this study, two decolorization methods—AB-8 macroporous resin and H_2_O_2_—were used for decolorization. We found that the retention of polysaccharides was higher when the macroporous resin method was used, while the decolorization efficiency was higher when H_2_O_2_ was used. This may be because the two methods involve different mechanisms of decolorization. As water-insoluble solid polymers with a macroporous structure and a significant specific surface area, macroporous resins can selectively enrich organic materials from a solution through physical adsorption [31,32]. Meanwhile, H_2_O_2_ can chemically attack pigments after it ionizes and releases HO_2_^-^ in aqueous solutions. In alkaline media, the level of ionization increases, and the decolorization effect is strengthened [33]. Hence, by employing AB-8 macroporous resin and H_2_O_2_, we successfully developed two quick, dependable, and practical decolorization processes. To investigate the differences in the biological activities of the two RGPs, we performed subsequent experiments using an LPS-induced IPEC-J2 cell inflammation model.

The intestinal tract, which largely consists of intestinal epithelial cells and closely knit intercellular enterocytes, is the body’s first line of defense against orally ingested pathogens and toxins that enter the intestinal lumen [34]. However, a number of factors, including poor nutrition, bacterial infections, and weaning stress, can lead to the overproduction of ROS, causing oxidative damage to the intestinal epithelium [35]. In our study, after LPS induction, the intracellular ROS and MDA content increased significantly, while the SOD content showed a significant decrease. MDA is a major byproduct of ROS-induced membrane lipid peroxidation, and oxidative damage causes intracellular MDA buildup [36]. Subsequently, antioxidant defense systems, including CAT, SOD, and GSH-Px, are activated to protect cells from ROS-induced oxidative damage [37]. RGP treatment significantly reduced intracellular ROS and MDA levels while increasing the intracellular SOD content. Moreover, RGP-1-A was more protective than RGP-2-A, likely owing to the difference in the number of sulfate and uronic acid groups between these polysaccharides [20]. Meanwhile, the analysis of MMP revealed that LPS treatment led to a decrease in MMP, while RGP pretreatment inhibited this decrease and alleviated the damage caused by LPS. Our findings were consistent with those from Vasantharaja et al., who found that sulfated polysaccharides from seaweed can reduce intracellular ROS production and restore MMP in L-929 cells [38].

The Keap1-Nrf2 signaling pathway is a key regulator of resistance to OS. It not only serves as an important defense system against oxidative damage but is also a key enhancer of the body’s antioxidant capacity [39]. Under normal conditions, Nrf2 is anchored to the cytoplasm in an inactive state and shows low transcriptional activity. However, when cells are stimulated and OS is induced, Keap1 releases its inhibition of Nrf2 and promotes the uncoupling of the Nrf2-Keap1 dimer [40]. After uncoupling, Nrf2 translocates from the cytoplasm to the nucleus, binds to antioxidant response elements (AREs) on DNA, and initiates the transcription of downstream antioxidant stress kinase (SOD and CAT) and phase II detoxification enzyme (GST, NQO1, and HO-1) genes. It thus promotes the expression of antioxidant proteins and alleviates the damage caused by OS in intestinal epithelial cells [41,42]. In this study, LPS treatment decreased the expression of Nrf2 while increasing that of Keap1, thereby preventing the activation of the Nrf2/Keap1 pathway. This situation was reversed after RGP preconditioning. At the maximum polysaccharide concentration, RGP-1-A was more protective than RGP-2-A. Subsequent experiments showed that RGP treatment upregulated HO-1 and NQO1, but LPS treatment downregulated these genes. In a previous study, Thilina et al. used H_2_O_2_ to induce OS in Vero cells and found that sulfated Padina boryana polysaccharides significantly reduce intracellular ROS levels and exert anti-apoptotic effects while activating Nrf2/Keap1 signaling. The ability of this pathway to activate downstream elements such as CAT and SOD was also demonstrated [43]. Similarly, sulfated Cyclocarya paliurus polysaccharides were found to activate the Keap1/Nrf2 pathway and, thereby, protect dendritic cells from H_2_O_2_-induced damage while also reducing the content of MDA and increasing the contents of SOD and CAT [44]. 

The inflammatory response is a defining feature of ROS-induced OS because inflammatory components are involved in the chain of these metabolic events. Inflammatory response indicators include IL-1β, IL-6, and TNF-α. In the current study, we discovered that LPS treatment activates NF-κB and TLR4, which then induce the upregulation of genes associated with inflammation (IL-1β, TNF-α, and IL-6). This effect is greatly inhibited by RGP treatment, consistent with earlier observations [15,21]. In vivo studies of polysaccharides have also demonstrated their ability to relieve inflammation. Zhang et al. showed that dietary Glycyrrhiza polysaccharide supplementation can significantly increase the relative mRNA expression of sIgA, IL-10, TGF-β, and TLR-4 in weaned piglets and significantly downregulate the relative mRNA expression of IL-2 [45]. Additionally, Xie et al. demonstrated that jejunal p-NF-κB and total NF-κB levels in pigs are inhibited following the addition of Enteromorpha polysaccharides–zinc to feed, and intestinal inflammation-related cytokines such IL-6, IL-8, IL-12, and TNF-α are downregulated [46]. These results suggest that RGP can suppress the synthesis of pro-inflammatory cytokines while increasing the production of anti-inflammatory cytokines, thus attenuating the LPS-induced inflammatory response. 

The OS induced by ROS is closely related to apoptosis and causes damage and death in intestinal epithelial cells [47,48]. The main signaling routes for cell apoptosis and rupture are mediated by caspases [49]. Bax is an essential apoptotic protein, whereas Bcl-2 inhibits apoptosis [48]. The vulnerability of cells to apoptosis is regulated by a tunable modulator called the Bax/Bcl-2 ratio [50]. Interestingly, our findings suggest that RGP may protect IPEC-J2 cells from LPS-induced oxidative damage by decreasing the expression of key apoptosis-related genes upregulated by LPS.

Excessive ROS production induces increased intestinal permeability, which compromises the integrity of the intestinal epithelial barrier [51]. TJ proteins (claudins, occludin, and ZO-1) and mucoprotein (MUC-2) can strengthen intestinal barrier function by separating the internal environment from the external environment and preventing harmful substances from entering the body [52,53]. In this study, the relative expression of intracellular barrier-related mRNAs was significantly reduced after LPS induction. RGP significantly increased the levels of MUC-2, ZO-1, occludin, and claudin-1 in LPS-induced IPEC-J2 cells and enhanced their barrier function. Among the two polysaccharides, RGP-1-A had a superior protective effect on the intestinal barrier compared to RGP-2-A. This may be because RGP-1-A contains sulfate groups and has a negative charge on its molecular surface as it is composed of polyanions [54]. This can provide the negative charge needed to form biofilm channels, reducing charge loss, protecting the cell barrier, and enhancing resistance to external damage [55,56]. In previous studies, sulfated Astragalus polysaccharides were found to more effectively inhibit LPS-induced reductions in TJ proteins on Caco2 cells than un-sulfated Astragalus polysaccharides [56]. In vivo studies of polysaccharides have shown that they enhance intestinal barrier function. Wang et al. showed that dietary supplementation with *Astragalus* and ginseng polysaccharides increases the protein expression of occludin and claudin in the jejunum of LPS-treated piglets [57]. Similarly, supplementation with Enteromorpha polysaccharides–zinc improves the abundance of occludin in the jejunum and ileum and that of claudin-1 in the ileum in weaned piglets [46].

## 5. Conclusions

In summary, in this study, we have optimized two RGP decolorization strategies, the AB-8 macroporous resin method and the H_2_O_2_ method. Finally, we found that the retention of polysaccharides was higher when the macroporous resin was used, while the decolorization level of polysaccharides was higher when H_2_O_2_ was used. 

Subsequently, we used an LPS-induced oxidative damage model to explore the differences in the biological activities of the two RGP in IPEC-J2 cells. Our results indicate that the protective effect of RGP is mainly achieved through the activation of the Nrf2/Keap1 pathway, which upregulates downstream genes (HO-1 and NQO1), increases the activity of antioxidant enzymes (SOD), and, thereby, reduces intracellular ROS and MDA levels. In addition, RGP can relieve the intracellular inflammatory environment by reducing the expression of inflammatory cytokines, possibly by inhibiting the NF-κB/TLR4 pathway. In addition, RGP can enhance cell barrier function while reducing the expression of apoptotic genes to inhibit apoptosis. Furthermore, resin-depigmented RGP-1-A is more cytoprotective than H_2_O_2_-depigmented RGP-2-A, possibly because RGP-1-A contains higher levels of sulfuric acid and uronic acid groups, which enhance its biological activity. Although this study is limited to a cellular model, it provides new approaches and insights into reducing intestinal inflammation in piglets. In the future, we will conduct animal experiments in a piglet model to evaluate the role of RGP in vivo. 

## Figures and Tables

**Table 1:** Levels and codes of AB-8 macroporous resin decolorization variables used in the Box-Behnken design.

Factors	Coded	Coded Level		
		−1	0	1
AB-8 macroporous resin addition (%)	A	6	8	10
Temperature (°C)	B	40	50	60
Duration (min)	C	60	80	100
pH	D	4	5	6

**Table 2:** Levels and codes of H_2_O_2_ decolorization variables used in the Box-Behnken design.

Factors	Coded	Coded Level		
		−1	0	1
Temperature (°C)	A	45	50	55
Duration (h)	B	2	2.5	3
pH	C	8	9	10
H_2_O_2_ addition (%)	D	6	8	10

**Table 3:** Analysis of variance (ANOVA) for testing the fitness of the regression equation.

Source	Sum of Squares	Mean Square	F-Value	*p*-Value
Model	67.19	4.80	36.07	<0.0001
A	3.01	3.01	22.65	0.0005
B	0.9039	0.9039	6.79	0.0230
C	0.7928	0.7928	5.96	0.0311
D	4.41	4.41	33.13	<0.0001
AB	0.0050	0.0050	0.0379	0.8490
AC	0.4099	0.4099	3.08	0.1047
AD	4.77	4.77	35.85	<0.0001
BC	1.57	1.57	11.77	0.0050
BD	0.0776	0.0776	0.5834	0.4597
CD	9.28	9.28	69.71	<0.0001
A^2^	26.53	26.53	199.40	<0.0001
B^2^	16.64	16.64	125.05	<0.0001
C^2^	6.36	6.36	47.79	<0.0001
D^2^	25.50	25.50	191.64	<0.0001
Residual	1.60	0.1330		
Lack of Fit	1.49	0.1487	2.71	0.2995
Pure Error	0.1097	0.0549		
Correlation Total	68.79			
CV%	0.5404			
*R* ^2^	0.9768			
Adjusted-*R*^2^	0.9497			

A: AB-8 macroporous resin addition; B: temperature (°C); C: time (min); D: pH; CV%: coefficient of variation; *R*^2^: determination coefficient; Adjusted-*R*^2^: adjusted determination coefficient.

**Table 4:** Analysis of variance (ANOVA) for testing the fitness of the regression equation.

Source	Sum of Squares	Mean Square	F-Value	*p*-Value
Model	284.18	20.30	10.03	0.0001
A	5.07	5.07	2.51	0.1394
B	101.73	101.73	50.27	<0.0001
C	2.70	2.70	1.33	0.2705
D	16.90	16.90	8.35	0.0136
AB	2.46	2.46	1.22	0.2914
AC	4.81	4.81	2.38	0.1491
AD	0.2652	0.2652	0.1310	0.7236
BC	0.0992	0.0992	0.0490	0.8285
BD	7.37	7.37	3.64	0.0805
CD	12.46	12.46	6.16	0.0289
A^2^	81.89	81.89	40.46	<0.0001
B^2^	61.05	61.05	30.17	0.0001
C^2^	68.66	68.66	33.92	<0.0001
D^2^	37.81	37.81	18.68	0.0010
Residual	24.29	2.02		
Lack of Fit	15.09	1.68	0.5471	0.7877
Pure Error	9.19	3.06		
Correlation Total	308.47			
CV%	1.77			
*R* ^2^	0.9213			
Adjusted-*R*^2^	0.8294			

A: temperature (°C); B: time (h); C: pH; D: H_2_O_2_ addition (%); CV%: coefficient of variation; *R*^2^: determination coefficient; Adjusted-*R*^2^: adjusted determination coefficient.

## Data Availability

The data presented in this study are available on request from the corresponding author.

## Supplementary Materials

The following supporting information can be downloaded at: https://www.mdpi.com/article/10.3390/antiox12040914/s1, Figure S1 Response surface and contour plots showing the interaction of different variables affecting the decolorization of RGP with AB-8 macroporous resin. Figure S2 Response surface and contour plots showing the interaction of different variables affecting the decolorization of RGP with H_2_O_2_. Table S1 Box-Behnken design with response values for AB-8 macroporous resin decolorization comprehensive scores. Table S2 Box-Behnken design with response values for H_2_O_2_ decolorization comprehensive scores. Table S3 Primer sequences used for quantitative real-time PCR. Table S4 Program for quantitative real-time PCR.
