# Supplementary material for: Rehmannia glutinosa Polysaccharides: Optimization of the Decolorization Process and Antioxidant and Anti-Inflammatory Effects in LPS-Stimulated Porcine Intestinal Epithelial Cells
Source: Antioxidants (Basel). 2023 Apr 12;12(4):914. doi: 10.3390/antiox12040914 (PMC10136223; doi:10.3390/antiox12040914)
Supplement: Supplementary file 1 [file antioxidants-12-00914-s001.zip › antioxidants-2249482-supplementary.pdf]

Supplementary data

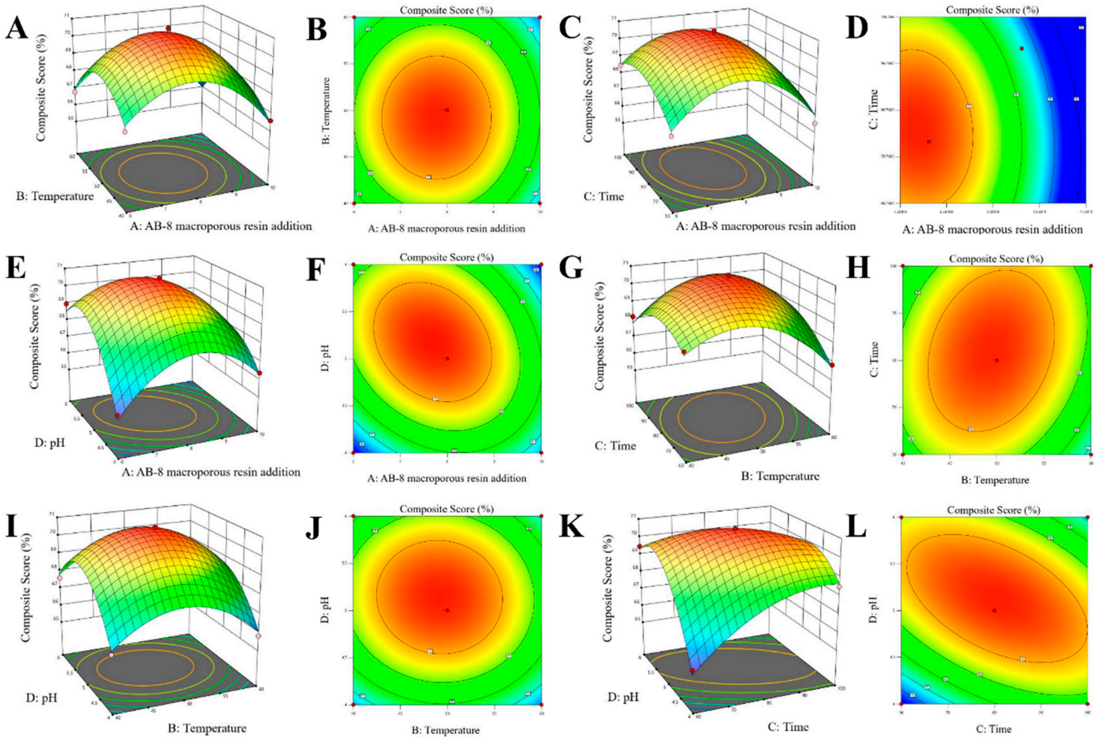

Figure S1 Response surface and contour plots showing the interaction of different variables affecting the decolorization of RGP using AB-8 macroporous resin.

n

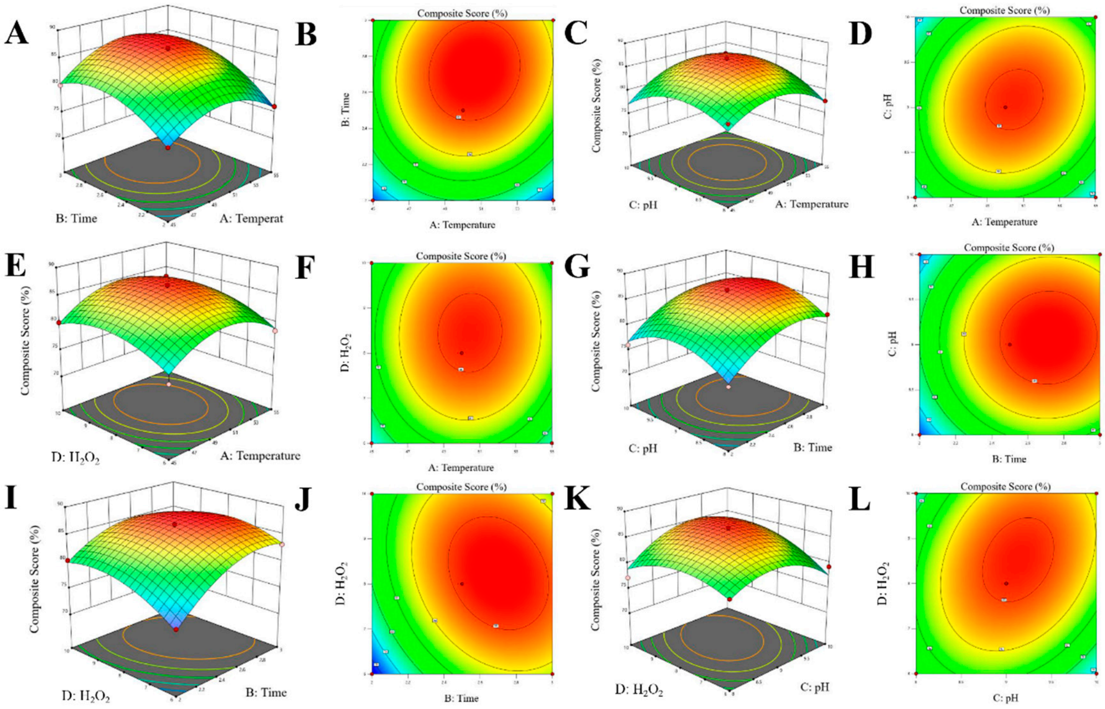

Figure S2 Response surface and contour plots showing the interaction of different variables affecting the decolorization of RGP using H_2O_2.

Table S1 Box–Behnken design with response values for comprehensive scores.

Group	A	B	C	D	DR (%)	RR (%)	CS (%)
1	0	-1	1	0	69.64	66.67	68.16
2	1	-1	0	0	60.52	72.69	66.61
3	0	-1	0	-1	95.24	37.02	66.13
4	0	-1	-1	0	70.75	66.67	68.71
5	-1	-1	0	0	73.34	61.19	67.27
6	0	-1	0	1	63.26	71.90	67.58
7	-1	0	0	-1	67.10	63.69	65.39
8	1	0	0	-1	70.14	62.59	66.37
9	0	0	-1	1	69.05	69.85	69.45
10	-1	0	-1	0	56.77	77.44	67.10
11	1	0	1	0	61.73	71.50	66.62
12	0	0	0	0	68.05	72.97	70.51
13	0	0	0	0	68.02	73.43	70.73
14	0	0	0	0	69.22	72.72	70.97
15	0	0	-1	-1	80.53	50.09	65.31
16	-1	0	1	0	60.80	76.13	68.47
17	0	0	1	-1	78.03	58.93	68.48
18	1	0	0	1	56.73	74.39	65.56
19	-1	0	0	1	46.89	91.02	68.95
20	0	0	1	1	71.11	61.94	66.53
21	1	0	-1	0	51.01	82.06	66.54
22	0	1	0	-1	81.33	50.27	65.80
23	1	1	0	0	84.73	47.77	66.25
24	0	1	1	0	77.53	60.07	68.80
25	-1	1	0	0	74.13	59.41	66.77
26	0	1	0	1	67.97	65.41	66.69
27	0	1	-1	0	63.34	70.35	66.85

A: AB-8 macroporous resin addition; B: Temperature (°C); C: Duration (min); D: pH; DR: Decolorization Rate (%); RR: Retention Rate (%); CS: Composite Score (%).

Table S2 Box–Behnken design with response values for comprehensive scores.

Group	A	B	C	D	DR (%)	RR (%)	CS (%)
1	-1	-1	0	0	85.5	66.98	76.24
2	-1	1	0	0	89.8	70.34	80.07
3	1	-1	0	0	85.33	66.85	76.09
4	1	1	0	0	93.15	72.97	83.06
5	0	0	-1	-1	89.91	70.43	80.17
6	0	0	-1	1	88.82	69.58	79.2
7	0	0	1	-1	86.51	67.77	77.14
8	0	0	1	1	93.34	73.12	83.23
9	-1	0	0	-1	89.76	70.32	80.04
10	-1	0	0	1	87.87	68.83	78.35
11	1	0	0	-1	87.07	68.21	77.64

12	1	0	0	1	92.59	72.53	82.56
13	0	-1	-1	0	84.31	66.05	75.18
14	0	-1	1	0	90.03	70.53	80.28
15	0	1	-1	0	93.21	73.01	83.11
16	0	1	1	0	92.84	72.72	82.78
17	-1	0	-1	0	85.67	67.11	76.39
18	-1	0	1	0	89.84	70.38	80.11
19	1	0	-1	0	87.95	68.89	78.42
20	1	0	1	0	93.28	73.06	83.17
21	0	-1	0	-1	84.59	66.27	75.43
22	0	-1	0	1	85.22	66.76	75.99
23	0	1	0	-1	91.93	72.01	81.97
24	0	1	0	1	93.26	73.06	83.16
25	0	0	0	0	96.61	75.67	86.14
26	0	0	0	0	97.37	76.27	86.82
27	0	0	0	0	96.55	75.63	86.09

A: Temperature (°C); B: Duration (h); C: pH; D: H_2O_2 addition (%); DR: Decolorization Rate (%); RR: Retention Rate (%); CS: Composite Score (%).

Table S3 Primer sequences used in quantitative real-time polymerase chain reaction.

Gene		Primer sequence 5′-3′	PCR product size (bp)	Gene bank accession number	E
GAPDH	Forward primer	GGAAGCTGTGGCGTGATGGC	175	NM_001206359.1	1.97
	Reverse primer	TTCTCCAGGCGGCAGGTCAG			
Bax	Forward primer	GGTCGCGCTTTTCTACTTTGCC	89	XM_013998624.2	1.97
	Reverse primer	TCCAGCCCATGATGGTCCT			
Bcl-2	Forward primer	CCAAGATCATTCTGACGGAGT	229	XM_021099593.1	2.00
	Reverse primer	GGTATCATAAGCCAGCAACGAA			
caspase-3	Forward primer	GGAATGGCATGTCGATCTGGT	351	NM_214131.1	2.01
	Reverse primer	ACTGTCCGTCTCAATCCCAC			

caspase-8	Forward primer	TCTGCGGACTGGATGTGATT	165	XM_021074714.1	1.93
	Reverse primer	TCTGAGGTTGCTGGTCACAC			
caspase-9	Forward primer	TGGAACTCAAGCCAGAGGAG	195	XM_013998997.2	1.92
	Reverse primer	CTGCATTCAGGACGTAAGCC			
Nrf2	Forward primer	CCAATTCAGCCAGCACAACACATC	149	XM_021075133.1	1.92
	Reverse primer	GACTGAGCCTGGTTAGGAGCAATG			
Keap1	Forward primer	GGAGGACCACACCAAGCAAGC	142	NM_001114671.1	1.98
	Reverse primer	GGATGAAGCCAGCACCACCTTG			
NQO1	Forward primer	GATCATACTGGCCCACTCCG	200	NM_001159613.1	1.95
	Reverse primer	GAGCAGTCTCGGCAGGATAC			
HO-1	Forward primer	TTGTGTCTCGTGTTTCCGTCT	112	NM_001004027.1	1.97
	Reverse primer	CCCCTCACCCCACCTTGCT			
TNF-α	Forward primer	TTCCAGCTGGCCCCTTGAGC	146	NM_214022.1	1.90
	Reverse primer	GAGGGCATTGGCATACCCAC			
IL-6	Forward primer	TTCACCTCTCCGGACAAAAC	122	NM_001252429.1	1.93
	Reverse primer	TCTGCCAGTACCTCCTTGCT			
IL-1β	Forward primer	GAGCTGAAGGCTCTCCACCTC	87	NM_001302388.2	1.91
	Reverse primer	ATCGCTGTCATCTCCTTGCAC			

NF-κB	Forward primer	AGTACCCTGAGGCTATAACTCGC	133	NM_001114281.1	2.04
	Reverse primer	TCCGCAATGGAGGAGAAGTC			
TLR4	Forward primer	CAGATAAGCGAGGCCGTCATT	113	NM_001293316.1	1.97
	Reverse primer	TTGCAGCCCACAAAAAGCA			

Table S4 Program for real-time fluorescent quantitative PCR (RT-PCR)

Reaction stage	Specific response	No. of cycles	Temperature	Duration
1	Predegeneration	1	95℃	30 s
2	Cyclic reaction	40	95℃	10 s
			60℃	30 s
3	Melting curve	1	95℃	15 s
			60℃	60 s
			95℃	15 s
